# Effects of an early rehabilitation program for adult cystic fibrosis patients during hospitalization: a randomized clinical trial

**DOI:** 10.1590/1414-431X2023e12752

**Published:** 2023-08-14

**Authors:** J. Flores, B. Ziegler, D. Silvello, P.T.R. Dalcin

**Affiliations:** 1 Programa de Pós-Graduação em Ciências Pneumológicas, Universidade Federal do Rio Grande do Sul, Porto Alegre, RS, Brasil; 2 Hospital de Clínicas de Porto Alegre, Serviço de Pneumologia, Universidade Federal do Rio Grande do Sul, Porto Alegre, RS, Brasil; 3 Programa de Pós-Graduação em Ciências Pneumológicas, Hospital de Clínicas de Porto Alegre, Serviço de Pneumologia, Universidade Federal do Rio Grande do Sul, Porto Alegre, RS, Brasil

**Keywords:** Cystic fibrosis, Adults, Physical exercise, Pulmonary exacerbation, Hospitalization

## Abstract

There is little information on pulmonary rehabilitation in patients with cystic fibrosis (CF) with pulmonary exacerbation. This study aimed to evaluate the effects of an early rehabilitation program on lung function, muscle strength, inflammatory markers, and quality of life in adults with CF hospitalized for pulmonary exacerbation. In this randomized controlled trial, 19 patients were included in the intervention group and 15 in the control group. The intervention group underwent an early rehabilitation program for 14 days after admission. All patients underwent spirometry, one-repetition maximum tests (1RM), and the 6-min walk test, and answered the Revised Cystic Fibrosis Questionnaire (CFQ-R) for quality of life and the International Physical Activity Questionnaire. Serum levels of interleukin and tumor necrosis factor alpha (TNF-α) were measured. In the intervention group, there were increases in 1RM biceps (P=0.009), triceps (P=0.005), shoulder abductors (P=0.002), shoulder flexors (P=0.004), hamstrings (P<0.001), and quadriceps values (P<0.001). In addition, there were improvements in CFQ-R-emotion (P=0.002), treatment burden (P=0.002), vitality (P=0.011), and physical scores (P=0.026), and a reduction in the Borg resting fatigue score (P=0.037). The interleukins levels did not change after the intervention. In adult CF patients with pulmonary exacerbation, early hospital rehabilitation had a significant impact on improving resting fatigue, muscle strength, and quality of life.

## Introduction

Cystic fibrosis (CF) is a common autosomal recessive disorder among Caucasians caused by mutations in the gene that encodes the CF transmembrane conductance regulator. The disorder is characterized by chronic pulmonary infection, bronchiectasis, exocrine pancreatic insufficiency, and a high concentration of electrolytes in sweat (1). The main factor responsible for morbidity and mortality in CF patients is pulmonary disease (2).

A combination of progressive limitations in physical fitness and increasing inactivity in CF patients leads to a vicious cycle in which dyspnea worsens and physical strength decreases, leading to severe quality of life (QoL) impairment (3,4). The main causes of exercise intolerance are associated with reduced ventilatory capacity, altered ventilation/pulmonary perfusion ratio, loss of peripheral skeletal muscle mass, and decreased cardiovascular function. Exercise-limiting symptoms in CF patients include fatigue, dyspnea, bronchospasm, ventilatory limitation, and cardiac dysfunction (5,6).

Regular exercise in CF patients improves cardiorespiratory capacity, reduces lung function decline (6,7), increases maximal oxygen uptake and peak oxygen uptake, reduces exercise-induced lactic acid production, and increases the oxidative capacity of skeletal muscles, as well as improving psychological factors such as self-esteem, self-fulfillment, self-confidence, and QoL (8,9).

Regular exercise combined with standard therapy has resulted in improved mucociliary clearance, body composition, bone development, immune function (10), and inflammatory cytokines levels (6,7). Moreover, it decreases insulin resistance, protein degradation, and resting heart rate in these patients (10).

Pulmonary exacerbation is the leading cause of hospitalization in individuals with CF, and during hospitalization, their physical activity level, exercise tolerance, and muscle strength decrease (11). However, few studies have been published on physical exercise in CF patients hospitalized for pulmonary exacerbation (10).

The main objective of this study was to evaluate the effects of an early rehabilitation program involving aerobic and muscle strength training in adult CF patients admitted to the Hospital de Clínicas de Porto Alegre (HCPA) due to exacerbation of lung disease.

## Material and Methods

This randomized controlled trial was approved by the HCPA Research Ethics Committee (15-0443) and was registered in the Clinical Trials platform (NCT03100214). All patients gave consent prior to inclusion.

The study included patients diagnosed with CF according to consensus criteria (12). All patients were over 18 years of age and were regularly followed up in the HCPA's Program for Adolescents and Adults with CF.

Exclusion criteria were cardiac, orthopedic, or trauma complications that precluded exercise performance, pregnancy, hemodynamic instability, massive hemoptysis, pneumothorax, and continuous use of noninvasive ventilation.

Initially, general information such as gender, age, age at diagnosis, body mass index (BMI), and bacteriology were collected. Then, the Cystic Fibrosis Questionnaire Revised (CFQ-R) (13) and the International Physical Activity Questionnaire (IPAQ) (14) were applied.

After the interview and initial data collection, the patients underwent the following evaluations: spirometry, maximal one repetition test (1RM), the six-minute walk test (6MWT), and a C-reactive protein (CRP) assay. These evaluations were performed within 24 h of admission and again on the 14th day of hospitalization.

Spirometry was performed at the Pulmonary Physiology Unit of the HCPA Pulmonology Service with a Jaeger 4.31a spirometer (Germany) according to the Brazilian Society of Pulmonology and Phthisiology technical criteria (15). Forced expiratory volume in one second (FEV_1_), forced vital capacity (FVC), and the FEV_1_/FVC ratio were measured. Results are reported in liters and as a percentage of the predicted value for sex, age, and height (15).

Functional capacity assessment was performed at the HCPA Pulmonology Service using the 6MWT according to the American Thoracic Society and European Respiratory Society guidelines (16). At the beginning and the end of the test, data on heart rate, respiratory rate, peripheral oxygen saturation (SpO_2_), and blood pressure were recorded, and the Borg scale was used to score dyspnea and leg fatigue; SpO_2_ was monitored throughout the test.

To measure muscle strength, the 1RM test was used, i.e., the maximum amount of weight that the patient could lift once during a standardized weightlifting exercise (17). The patients underwent the 1RM test for the following muscle groups: biceps, triceps, shoulder abductors, shoulder flexors, hamstrings, and quadriceps.

Peripheral venous blood samples were collected from all subjects in coated tubes. Blood samples were centrifuged for 15 min at 4°C and 302 *g* within 1 h from collection. Serum was separated, aliquoted into 1.5-mL microtubes and stored at -80°C for further analysis.

Serum levels of inflammatory cytokines interleukin (IL)-1, IL-6, IL-10, IL-17, and tumor necrosis factor (TNF)-α were measured by Multiplex Bead Immunoassay, using Human Magnetic Custom Luminex Kit by the Luminex system 200 (Invitrogen by Life Technologies, USA). All analyses were performed in duplicate using commercial assays and in accordance with the manufacturer's recommendations.

Within 48 h of hospitalization, the patients were randomized to either the intervention or control group. Randomization was performed with the Research Randomizer (http://www.randomizer.org/form.htm) in blocks of 6 patients by an individual not otherwise involved in the study, who kept this procedure confidential. After electronic randomization, the results were placed in brown paper envelopes according to order of inclusion. In each case, the envelope was only opened after the patient had completed the entire initial evaluation.

The exercise group, besides receiving the same monitoring as the control group, also received physical training through the early rehabilitation program, which began within 48 h of hospitalization. The patients underwent supervised physical training 5 times per week for 14 days, with sessions lasting between 45 min and 1 h. The individual who performed the outcome assessments was blinded to group allocation.

The aerobic exercise intensity was based on the 6MWT results, kept within a target range of 80% of the test speed. The exercise was performed on a treadmill with a desired duration of 30 min.

The intensity of anaerobic exercise was calculated based on the 1RM test results, kept within a range of 30-40% of the maximum load. Patients performed three sets of 10 repetitions for the following muscle groups: biceps, triceps, shoulder abductors, shoulder flexors, hamstrings, and quadriceps.

Patients randomized to the control group continued to be monitored by the entire team from the HCPA Program for Adolescents and Adults with CF (physician, nutritionist, psychologist, and physiotherapist) throughout their hospitalization. Supervised physiotherapy included respiratory therapy, which involved inhalation therapy and secretion removal techniques.

SPSS version 22.0 (IBM, USA) was used to process and analyze the data. A descriptive analysis of the study variables in each group was performed. Quantitative data are reported as means±SD or median (interquartile range). Qualitative data are reported as n (% of all cases). For the comparison between groups, a *t*-test for paired samples was used for normally distributed continuous variables, while the Mann-Whitney U test was used for non-normally distributed continuous variables. Qualitative data were analyzed using the chi-squared test with Yates correction or Fisher's exact test, as necessary. For analysis of repeated measures at baseline and on the 14th day of hospitalization, a generalized estimating equation model (group- and moment-based approach) was used. The analysis was performed by intention-to-treat method and, in case of missing data, the baseline value was repeated. All statistical tests were two-tailed, and the significance level was set at 5%. Kaplan-Meier graphs were used to demonstrate survival over time. Patients who underwent lung transplantation were censored at the time of their operation, and patients who were alive were censored at the “end of the study period”. Kaplan-Meier survival analysis was used to calculate the time to a new hospitalization 90 after days of discharge.

The sample size was calculated following Ziegler et al. (18). As a reference value for distance covered in the 6MWT, an expected effect magnitude of 50 meters was used, with a standard deviation of 70 meters, for a bidirectional alpha of 0.05 and a power of 80%. The estimated sample size was 34 patients in each group.

## Results

**Figure 1:**
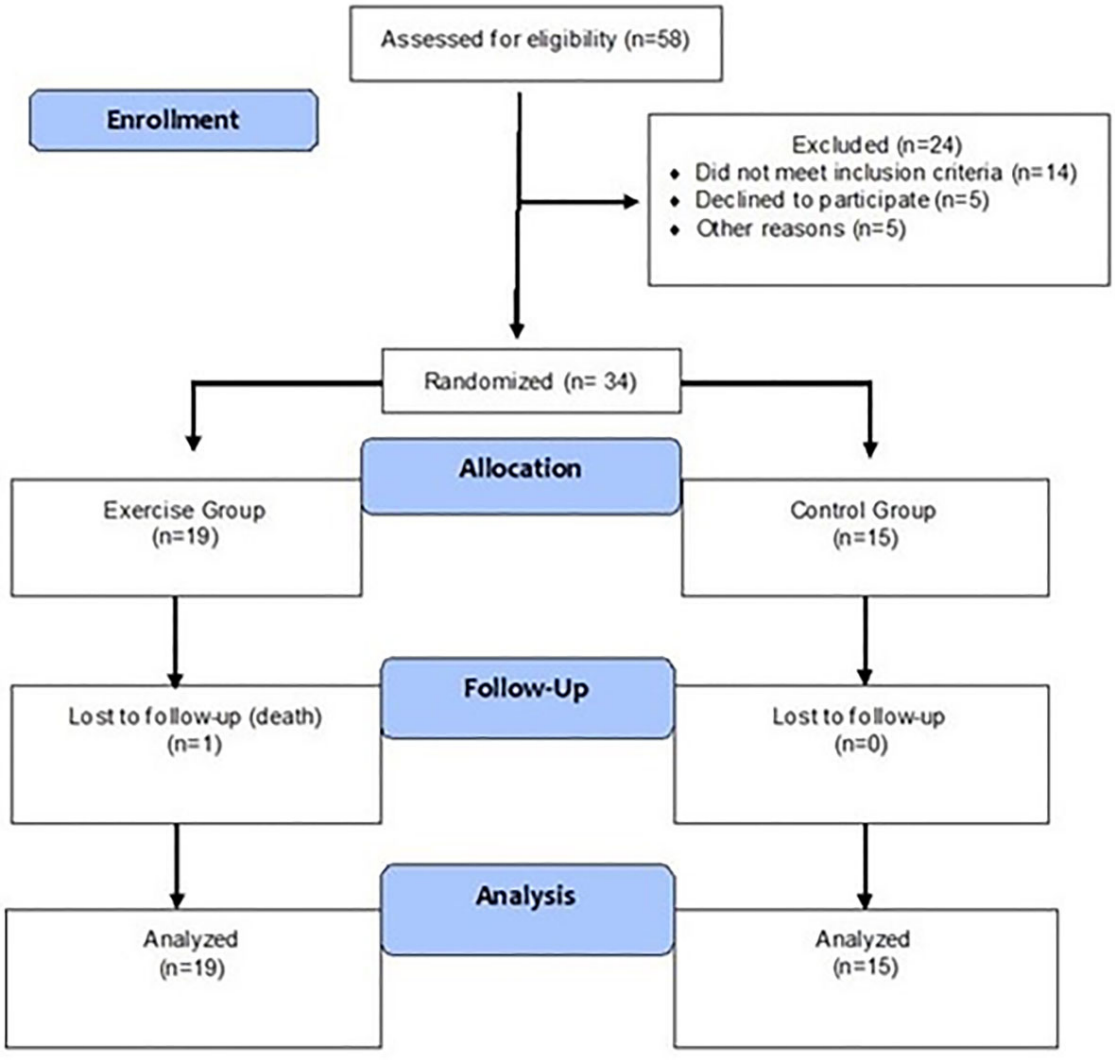
Study flow diagram.

From August 2016 to September 2018, 58 CF patients were hospitalized at the HCPA for pulmonary exacerbation (Figure 1). Of these, 24 were excluded: five were already undergoing pulmonary rehabilitation and chose to continue their own program; five remained in the emergency room for more than 48 h, which precluded inclusion in the study; the condition of two was very severe and required continuous noninvasive ventilation, so they could not participate; two had massive hemoptysis; five refused to participate in the study; and five for other reasons (1 had superior vena cava stenosis, 2 pregnancies, 1 was hospitalized for lung transplantation, and 1 had sepsis). Thus, 34 patients were included, 19 were randomized to the exercise group and 15 were randomized to the control group.

The general characteristics of the patients included in the study are described in Table 1. No significant differences in baseline clinical characteristics and pulmonary function were observed between the two groups.

**Table 1:** Characteristics of the control and exercise groups.

Variables	n=34	Exercise group (n=19)	Control group (n=15)	P
Gender, n (%)				
Female	21 (61.8)	12 (63.2)	9 (60.0)	1.00
Male	13 (38.2)	7 (36.8)	6 (40.0)	1.00
Age (years), median (IQR)	29 (12)	29 (12)	27 (13)	0.531
Age at diagnosis (years), median (IQR)	2 (8.8)	2 (16.7)	1 (6.0)	0.306
Body mass index (kg/m^2^), mean±SD	20.4±2.1	21.0±2.1	19.7±2.1	0.089
Microbiology, n (%)				
*Pseudomonas aeruginosa*	26 (76.5)	15 (78.9)	11 (73.3)	1.00
MSSA	17 (50.0)	7 (36.8)	10 (66.7)	0.166
MRSA	8 (23.5)	3 (15.8)	5 (33.3)	0.417
*Burkholderia cepacia*	5 (14.7)	4 (21.1)	1 (6.7)	0.355
FEV_1_ (% pred), mean±SD	36.0±12.2	36.6±13.6	35.3±10.5	0.770
FVC (% pred), mean±SD	50.2±15.4	49.7±15.9	50.8±15.3	0.834
FEV_1_/FVC (% pred), mean±SD	71.6±9.4	61.5±7.0	60.2±10.1	0.652
6MWT, mean±SD				
SpO_2_ rest (%), mean±SD	92.9±3.2	92.4±3.3	93.5±3.0	0.350
SpO_2_ end (%), mean±SD	87.7±6.5	87.0±6.9	88.5±5.9	0.500
Distance (m), mean±SD	485.9±99.1	466.1±107.2	510.9±84.6	0.194
IPAQ (MET), median (IQR)	1247.0 (3008)	1798.0 (2467)	1074.0 (4041)	0.986

SD: standard deviation; IQR: interquartile range; MSSA: methicillin-susceptible *Staphylococcus aureus*; MRSA: methicillin-resistant *Staphylococcus aureus*; 6MWT: six-minute walk test; FEV_1_: forced expiratory volume in the first second; FVC: forced vital capacity, SpO_2:_ peripheral oxygen saturation; IPAQ: International Physical Activity Questionnaire; MET: metabolic equivalent of task. *t*-test or Mann-Whitney U test and chi-squared test or Fisher's exact test.

**Figure 2:**
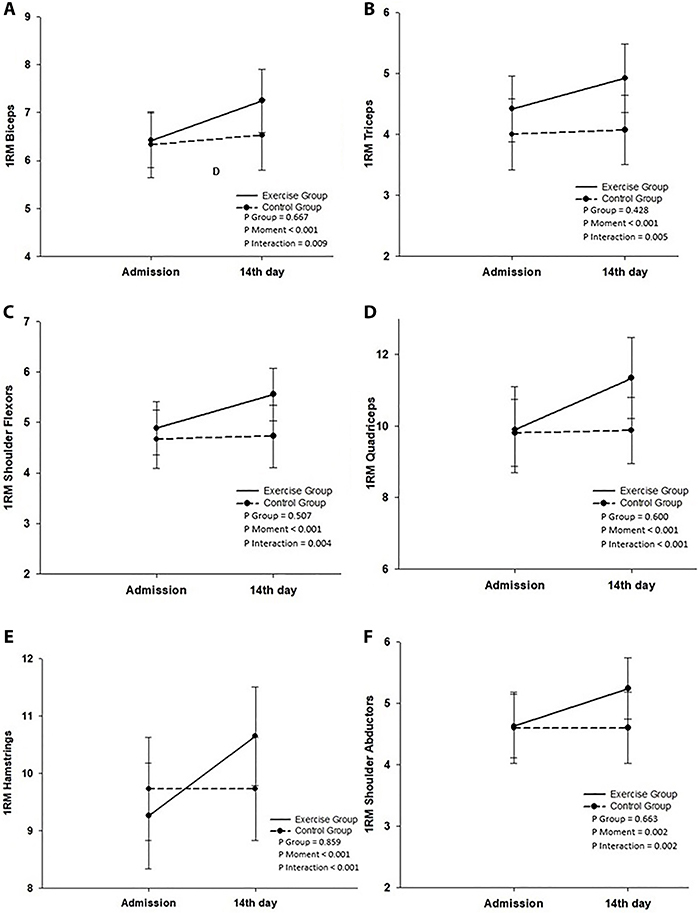
Repeated measures analysis using a generalized estimating equation for muscle strength measurement in the maximal repletion test (1RM) for biceps (**A**), triceps (**B**), shoulder flexors (**C**), quadriceps (**D**), hamstrings (**E**), and shoulders abductors (**F**). Data are reported as means±SD. The Borg score for resting fatigue was significantly lower (P=0.037) in the intervention group than the control group.

**Figure 3:**
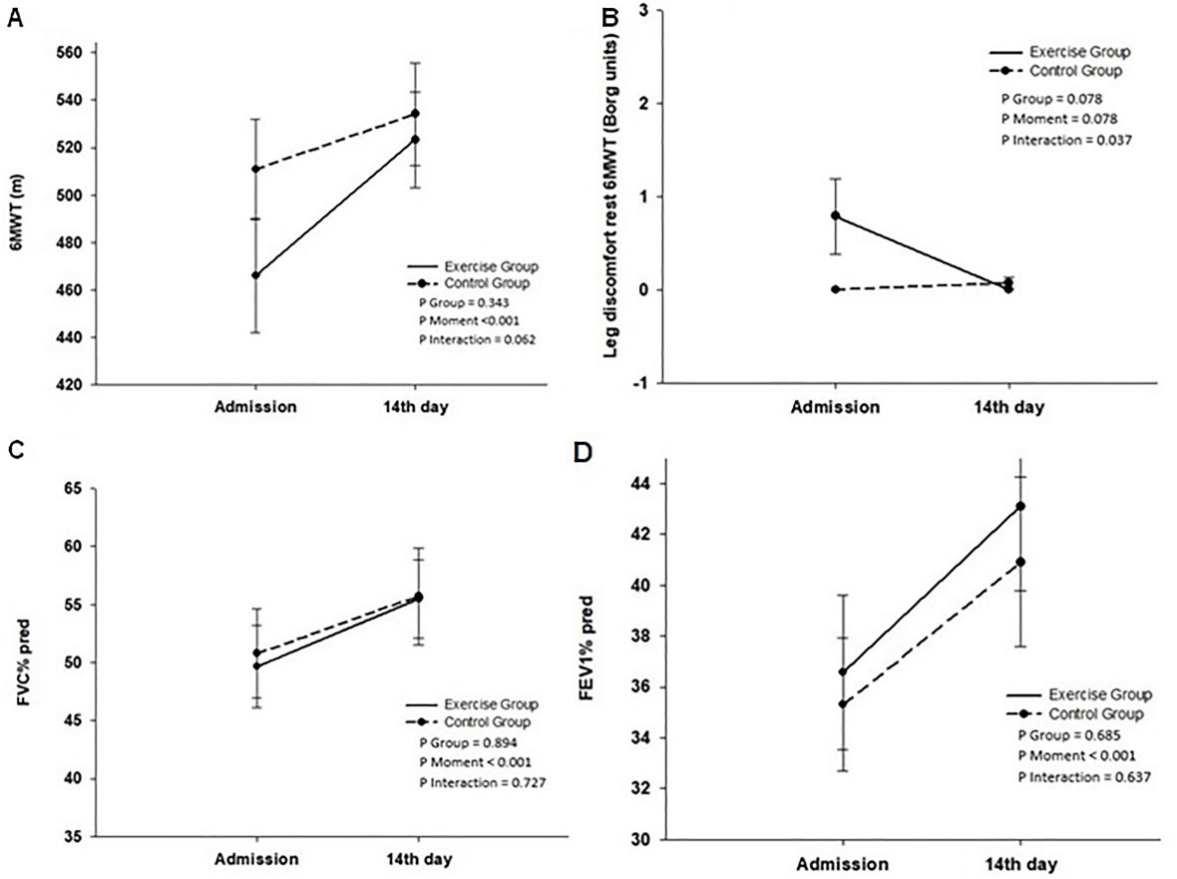
Repeated measures analysis using a generalized estimating equation for distance covered in the six-minute walk test (6MWT) (**A**), Borg fatigue score (**B**), forced vital capacity as % of predicted (FVC% pred) (**C**), and forced expiratory volume in one second as % of predicted (FEV1% pred) (**D**). Data are reported as means±SD.

At the end of the rehabilitation program period, in the exercise group there were significant increases in muscle strength in biceps (P=0.009), triceps (P=0.005), shoulder abductors (P=0.002), shoulder flexors (P=0.004), hamstrings (P<0.001), and quadriceps (P<0.001) demonstrated by the 1RM test compared to the control group and baseline measurements (Figure 2). Nonetheless, no significant differences were observed between groups in the distance covered in the 6MWT test (P=0.062), FEV_1_ (P=0.637), and FVC (P=0.727) (Table 2, Figure 3).

**Table 2:** Exercise capacity, lung function, and quality of life at admission and on the 14th day of hospitalization.

Variable	Exercise group (n=19)		Control group (n=15)		P interaction
	Admission	14th day	Admission	14th day	
Distance walked (m), mean±SD	466±107	523±92**	511±85	534±87	0.062
SpO_2_ rest (%), mean±SD	92±3	94±4	93±3	94±2	0.370
SpO_2_ end 6MWT (%), mean±SD	87±7	88±7	89±6	88±7	0.231
Borg dyspnea rest, mean±SD	0.9±2.0	0.0±0.0	0.3±0.7	0.1±0.3	0.176
Borg dyspnea end 6MWT, mean±SD	3.4±2.7	2.3±2.3	2.9±3.0	2.2±2.9	0.521
Borg fatigue rest, mean±SD	0.8±1.8	0.0±0.0	0.0±0.0	0.1±0.3	0.037
Borg fatigue end 6MWT, mean±SD	3.0±2.1	2.7±3.1	2.3±2.6	1.9±2.7	0.875
BMI (kg/m^2^), mean±SD	20.9±2.1	21.6±2.1**	19.7±2.1	20.5±2.0**	0.366
FEV_1_ (% pred), mean±SD	36.6±13.6	43.1±14.5**	35.3±10.5	40.9±13.4**	0.637
FVC (% pred), mean±SD	49.7±15.9	55.5±14.7**	50.8±15.3	55.7±16.7*	0.727
FEV_1_/FVC (% pred), mean±SD	72.7±8.3	76.2±9.9**	70.3±10.7	73.8±10.7**	0.989
CRP, mean±SD	53.3±74.2	7.5±8.0**	32.5±23.7	6.5±5.8**	0.262
1RM biceps, mean±SD	6.4±2.6	7.3±3.0	6.3±2.8	6.5±3.0	0.009
1RM triceps, mean±SD	4.4±2.5	5.0±2.6**	4.0±2.3	4.1±2.3	0.005
1RM shoulder flexors, mean±SD	4.9±2.4	5.7±2.4**	4.7±2.4	4.7±2.5	0.004
1RM shoulder abductors, mean±SD	4.6±2.4	5.3±2.3**	4.6±2.3	4.6±2.4	0.002
1RM quadriceps, mean±SD	9.9±5.4	11.5±5.2**	9.8±3.7	9.9±3.8	<0.001
1RM in hamstrings, mean±SD	9.3±4.1	10.9±3.9**	9.7±3.6	9.7±3.6	<0.001
CFQ-R-domain, mean±SD					
Body Image	77.8±22.2	81.5±17.9	58.5±28.0	65.9±25.0**	0.227
Digestive symptoms	90.1±9.7	94.5±7.8*	83.0±19.6	89.6±18.5	0.280
Eating	73.1±21.7	84.6±18.7**	76.2±29.3	85.2±18.6*	0.817
Emotional functioning	61.1±17.3	72.2±14.3**	70.7±14.2	72.0±19.9	0.002
Health perceptions	32.7±22.1	45.7±24.7**	43.7±25.0	48.9±20.1	0.202
Physical functioning	36.2±23.4	58.3±27.1**	53.6±31.0	61.7±25.3	0.026
Respiratory symptoms	38.0±16.8	61.7±17.0**	43.3±15.0	65.2±16.5**	0.734
Role functioning	57.0±21.2	55.1±24.6	68.4±21.9	67.8±22.5	0.996
Social functioning	57.6±16.9	57.4±18.5	64.5±13.2	64.5±14.8	0.996
Treatment burden	42.7±23.5	50.6±21.7**	61.5±18.7	54.8±19.5	0.002
Vitality, mean±SD	35.2±15.5	68.1±14.9**	53.3±19.1	73.3±16.7**	0.011
Weight, mean±SD	50.9±42.1	68.5±35.19	28.9±39.6	73.3±28.7**	0.054

6MWT: six-minute walk test; SpO_2_: peripheral oxygen saturation; BMI: body mass index; FEV_1_: forced expiratory volume in the first second; FVC: forced vital capacity; CRP: C-reactive protein; 1RM: one-repetition maximum strength test; CFQ-R: cystic fibrosis questionnaire revised. *P<0.05, **P<0.01 for differences between admission and the 14th day of hospitalization (paired *t-*test).

**Figure 4:**
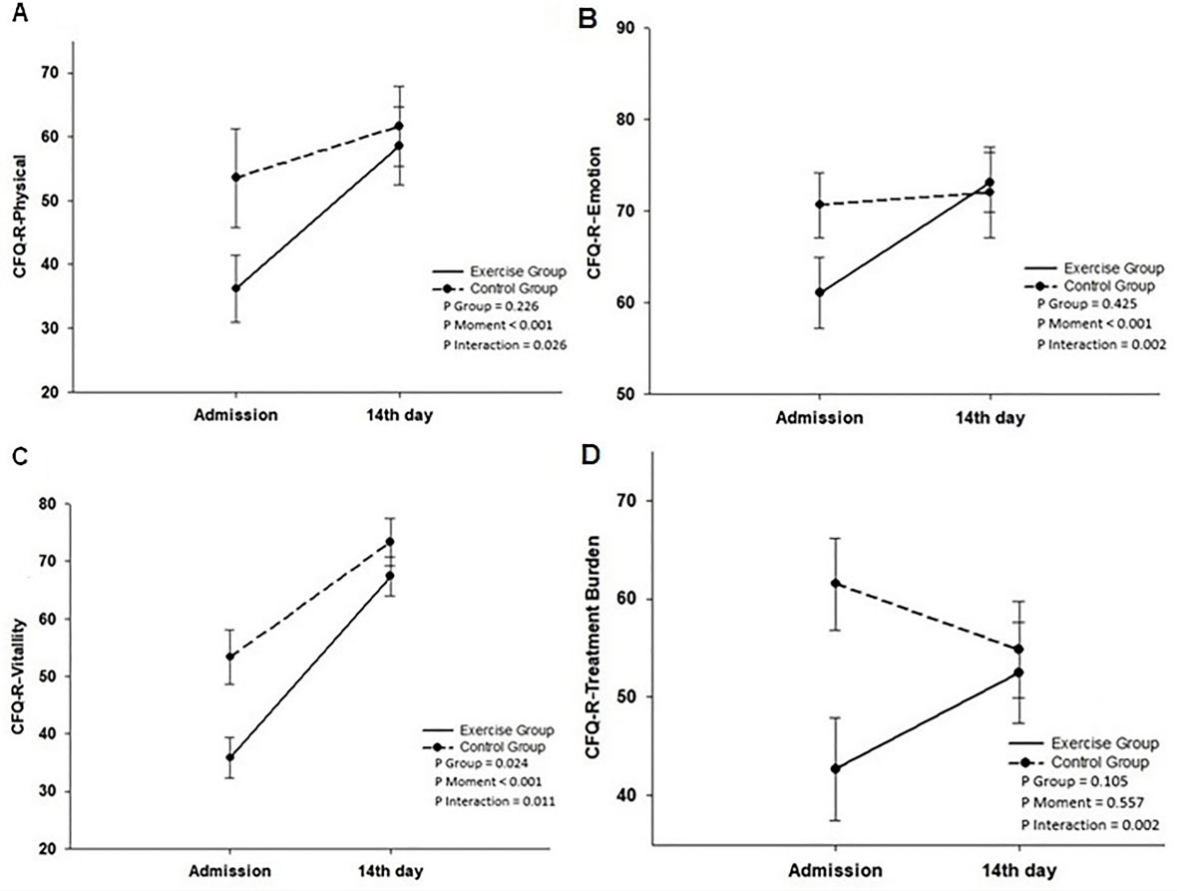
Repeated measures analysis using a generalized estimating equation for quality of life (QoL) scores. There were significant increases in the intervention group for the group × time interaction for the revised cystic fibrosis quality of life (CFQ-R) subscales: CFQ-R-physical (P=0.026) (**A**), CFQ-R-emotion (P=0.002) (**B**), CFQ-R-vitality (P=0.011) (**C**), CFQ-R-treatment burden (P=0.002) (**D**). Data are reported as means±SD.

Additionally, exercise group patients showed improvement in the QoL scores for the emotional (P=0.002), physical (P=0.026), treatment (P=0.002), and vitality (P=0.011) domains compared to the control group and baseline data. Similarily, the Borg score for resting fatigue was significantly lower (P=0.037) in the intervention group than in the control group (Table 2, Figure 4).

**Figure 5:**
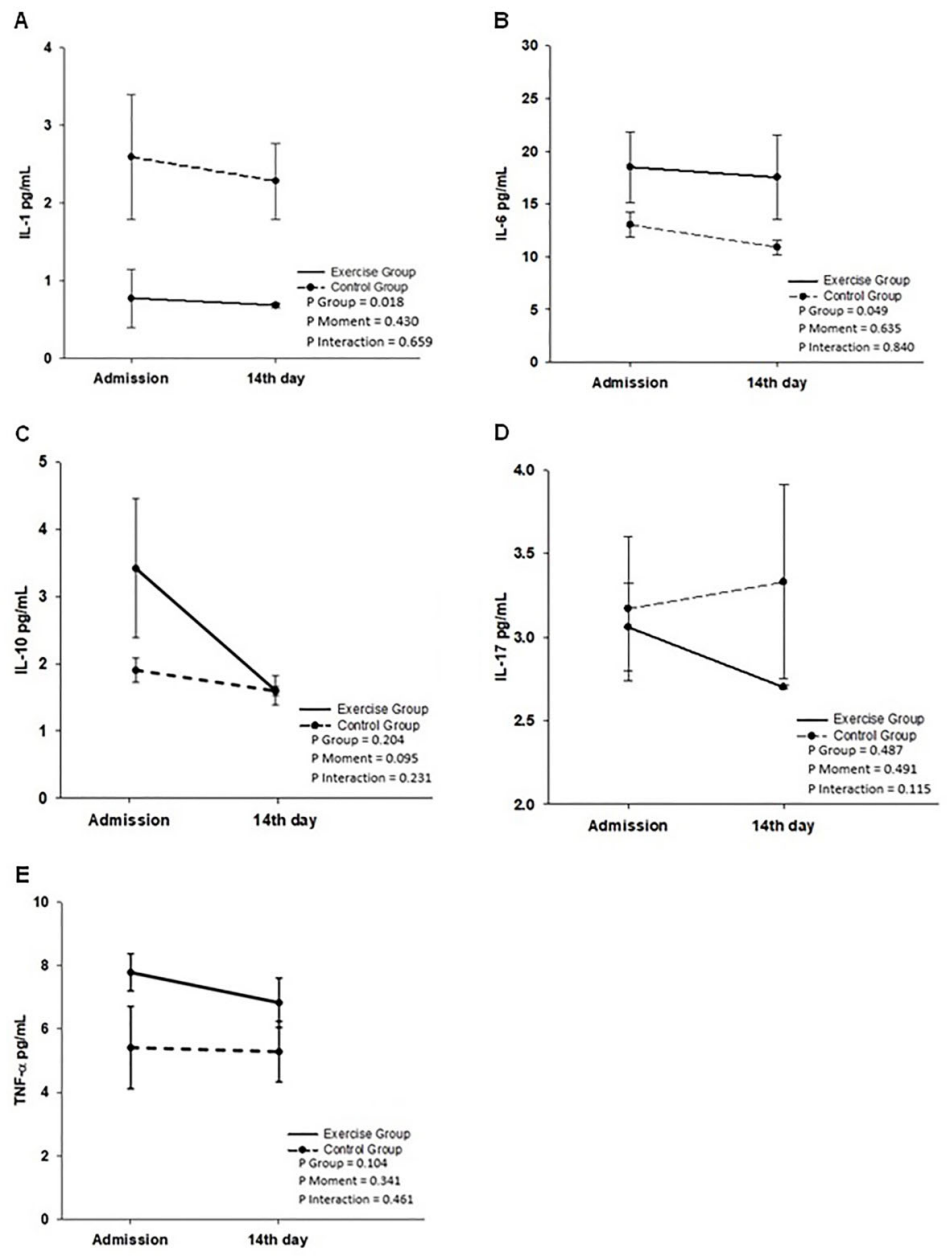
Interleukin (IL)-1 (**A**), IL-6 (**B**), IL-10 (**C**), IL-17 (**D**), and tumor necrosis factor (TNF)-α (**E**) serum levels in patients with cystic fibrosis in the exercise and control groups. Data are reported as means±SD. Statistical analysis was performed by generalized estimating equation with the Bonferroni *post hoc* test.

Unexpectedly, baseline IL-1 levels were significantly higher in the control group than in the exercise group (Figure 5A). Likewise, baseline IL-6 levels were lower in the control group (Figure 5B). These differences remained at the end of the study, demonstrating that the intervention did not change IL-1 and IL-6 levels (Figure 5A and B). Baseline levels of IL-10, IL-17, and TNF-α were similar between groups and did not show significant changes after the intervention (Figure 5C, D, and E).

We performed a Kaplan-Meier analysis for time to a new hospitalization at 90 days after discharge. Mean time to a new hospitalization was 87.4 days in the exercise group (95%CI: 84.0-90.8 days) and 79.5 days in control group (95%CI: 71.5-87.5 days), log-rank test P=0.107.

## Discussion

This randomized clinical trial evaluated the effects of an early rehabilitation program (aerobic and muscle strength training) on lung function, muscle strength, inflammatory markers, and quality of life in adult CF patients hospitalized for pulmonary exacerbation. Patients showed significant improvement in the Borg score for leg fatigue at rest, muscle strength variables (1RM biceps, 1RM triceps, 1RM shoulder flexors, 1RM shoulder abductors, 1RM quadriceps, and 1RM hamstrings), and CFQ-R domains (emotional functioning, physical functioning, social functioning, and treatment burden) after the rehabilitation program.

Studies on the effects of exercise during hospitalization in CF patients are scarce in the literature (6). Selvadurai et al. (10) studied 66 children and adolescents with CF aged 8 to 16 years who had been hospitalized for pulmonary exacerbation. They were randomized into three patient groups: 15 patients in an aerobic training group, 18 in an anaerobic training group, and 16 in a control group, with FEV_1_ of 56.8, 58.0, and 57.4%, respectively. Aerobic training of five 30-min sessions per week at an intensity of 70% peak heart rate was most effective in improving aerobic capacity and QoL. Strength training, performed in five sets of 10 repetitions with a 70% maximal load was best for FEV_1_ improvement and for gains in lean mass and leg strength. In the present study, no difference was identified between groups regarding pulmonary function variables. The short training time and the severity of the underlying disease are factors that may have influenced this result.

A recently published systematic review (19) assessed the effects of supervised pulmonary rehabilitation programs on exacerbated chronic obstructive pulmonary disease (CPOD) patients initiated during hospitalization or up to 4 weeks after discharge. Substantial benefits from early supervised pulmonary rehabilitation were observed in exacerbated COPD patients. The results of the meta-analysis showed that patients who underwent pulmonary rehabilitation at this stage had better prognosis, QoL, and distance covered in the 6MWT than the group that received normal care. Based on the included studies' moderate to low level of evidence, the authors concluded that pulmonary rehabilitation reduces the risk of mortality, hospital readmissions, and days of hospitalization and provides clinically relevant improvements in QoL and functional capacity.

In the present study, the exercise group showed improved QoL in the CFQ-R domains (emotional functioning, physical functioning, social functioning, and treatment burden). Adolescents and adults with CF have lower QoL and high rates of depression and anxiety (20). Several studies (7) conducted with CF patients have found that exercise provides QoL-related benefits, which corroborates our findings. Schmidt et al. (21) studied a population of CF patients over 12 weeks who exercised at least three times a week for 30 min. Significant QoL improvement was observed in the treatment burden and emotional functioning domains. Nevertheless, there is no consensus in the literature regarding the type of exercise, duration, load, and intensity necessary to promote such benefits.

In this study, the distance covered in the 6MWT did not significantly differ between groups. The 6MWT was selected as the main outcome assessment due to the critical condition of the patients. The exercise group did not differ from the control group, although it showed greater increments in the 6MWT result (57 *vs* 23 m). Possibly, the small sample size played a role in this result. The 6MWT is a valuable test for CF patients and those with severe disease since it is considered a predictor of morbidity and mortality and has a significant correlation with maximal oxygen uptake (VO_2_max) (22). VO_2_max, the maximum amount of oxygen that can be absorbed during exercise, has been used for assessing physical training in CF patients (23).

Using the cardiopulmonary exercise test, which is considered the gold standard for assessing the interaction of cardiovascular, respiratory, and metabolic systems (24), to assess maximal exercise capacity in this population might better detect short-term changes. However, its availability is very limited in our region, and it is more difficult to perform in patients with severe conditions. Selvadurai et al. reported that anaerobic training was not associated with improved VO_2_max compared to controls at discharge or one month after discharge (10).

There was a significant improvement in arm and leg muscle strength in the exercise group despite the short training period. Several studies have observed changes in muscle strength in CF patients after a training period (10,25-27). Some authors report that increased muscle strength in CF patients is directly related to improved exercise tolerance (3,28). Compared to the control group, the training group demonstrated significant increases in muscle strength. However, to date, there are no reference equations in the literature for calculating normality values and estimating the magnitude of the effect.

Our exercise rehabilitation program did not change any of the inflammatory markers analyzed. On the other hand, another study demonstrated that physical activity modulates IL-6 and TNF- levels in patients with CF (29). However, Nigro et al. (29) evaluated patients who performed supervised physical activity regularly for three years before the study. Similarly, other studies showed that brief and moderate exercise can improve inflammatory cytokines in CF (6,30). Compared to other studies, our intervention had short duration and included hospitalized patients on intensive therapy. This difference in method and study population may explain the unaltered inflammatory profile.

The clinical findings of the present study suggest that even supervised physical exercises performed in a short period of time and in an adverse situation of disease exacerbation can have positive impacts on muscle strength, fatigue, and quality of life.

This study had some limitations. First, due to technical impediments, it was not possible to reach the minimum sample size calculated for the main outcome, which could have underestimated the real impact of early rehabilitation in this group. In addition, the study was conducted at a single center. Furthermore, early rehabilitation was performed only during hospitalization and the outcome assessment time was short (14 days). Prolonged rehabilitation after hospital discharge and a longer time until outcome evaluation could shed more light on the subject. Moreover, it is not yet fully understood or established which form and intensity of aerobic and muscle strength training provide the maximum benefits for these patients.

In conclusion, the early rehabilitation program significantly improved the muscle strength, resting fatigue, and QoL in adult patients with CF.
